# Fatty acids-stress attenuates gluconeogenesis induction and glucose production in primary hepatocytes

**DOI:** 10.1186/1476-511X-11-66

**Published:** 2012-07-09

**Authors:** Noga Budick-Harmelin, Sarit Anavi, Zecharia Madar, Oren Tirosh

**Affiliations:** 1 School of Nutritional Sciences, Institute of Biochemistry, Food Science and Nutrition, The Robert H. Smith Faculty of Agriculture, Food and Environment, The Hebrew University of Jerusalem, Rehovot, 76100, Israel; 2 The Hebrew University of Jerusalem, P.O. Box 12, Rehovot, 76100, Israel

**Keywords:** Hypoglycemia, Liver, β-oxidation disorders

## Abstract

**Background:**

Hepatic gluconeogenesis tightly controls blood glucose levels in healthy individuals, yet disorders of fatty acids (FAs) oxidation are characterized by hypoglycemia. We studied the ability of free-FAs to directly inhibit gluconeogenesis, as a novel mechanism that elucidates the hypoglycemic effect of FAs oxidation defects.

**Methods:**

Primary rat hepatocytes were pre-treated with FAs prior to gluconeogenic stimuli with glucagon or dexamethasone and cAMP.

**Results:**

Pre-treatment with 1 mM FAs (mixture of 2:1 oleate:palmitate) for 1 hour prior to gluconeogenic induction, significantly decreases the induced expression of the gluconeogenic genes phosphoenolpyruvate carboxykinase (PEPCK) and glucose-6-phosphatase (G6pase) as well as the induced glucose production by the cells. The inhibitory effect of FAs upon gluconeogenesis is abolished when pre-treatment is elongated to 18 hours, allowing clearance of FAs into triglycerides by the cells. Replacement of palmitate with the non-metabolic fatty acid 2-bromopalmitate inhibits esterification of FAs into triglycerides. Accordingly, the increased exposure to unesterified-FAs allows their inhibitory effect to be extended even when pre-treatment is elongated to 18 hours. Similar changes were caused by FAs to the induction of peroxisome-proliferator-activated receptor-γ coactivator 1α (PGC1α) expression, indicating this transcriptional coactivator as the mediating link of the effect. This inhibitory effect of FAs upon gluconeogenic induction is shown to involve reduced activation of cAMP response element-binding (CREB) transcription factor.

**Conclusions:**

The present results demonstrate that free-FAs directly inhibit the induced gluconeogenic response in hepatocytes. Hence, high levels of free-FAs may attenuate hepatic gluconeogenesis, and liver glucose output.

## Background

The liver is a major site of systemic metabolic regulation. Pathways of synthesis and degradation of lipids and carbohydrates are controlled by complex interactions in the hepatocytes [1]. Induction of hepatic gluconeogenesis (GNG) promotes the synthesis and release of glucose into the blood, in order to maintain fasting normoglycemia among healthy individuals [2]. However, impaired glucose homeostasis, which leads to fasting-related hypoglycemia, is a prominent pathological feature of different fatty acids (FAs) oxidation disorders [3].

The main cellular site for oxidation of FAs is the mitochondria [4], where they are degraded in a cyclic process of β-oxidation into acetyl-CoA units. Disorders of mitochondrial FAs oxidation comprise a group of at least a dozen inherited defects of distinct enzyme or transporter deficiencies [5]. These diseases vary in their symptoms and severity, yet hypoglycemia is one major clinical sign in all FAs oxidation defects [6].

The pathogenic mechanisms leading to hypoglycemia in FAs oxidation defects have only partially been elucidated [7]. The observed disruption in carbohydrate management may result from combined effect of two processes. First, hypoglycemia can occur due to enhanced peripheral glucose uptake as an outcome of livers’ failure to produce ketone bodies [3]. Second, hypoglycemic response might reflect reduced GNG. In this regard, several different factors have been suggested to contribute to an insufficient hepatic glucose production among patients. Those include lack of acetyl-CoA which is a product of FAs oxidation and serves as allosteric activator of pyruvate carboxylase, high levels of non-esterified acyl-CoA esters that hamper activation of pyruvate carboxylase, and low levels of ATP and reducing equivalents (NADH) necessary for GNG [8,9].

The current study shades new light on the mechanistic basis of limited gluconeogenic capacity among patients with defects in FAs oxidation. It is demonstrated, for the first time, that FAs directly attenuate the induction of gluconeogenic programme in isolated primary hepatocytes and that removal of FAs restores the gluconeogenic response. Our findings indicate that free-FAs may be involved in inhibition of GNG induction, mediating hypoglycemia in FAs oxidation disorders.

## Results

### Free-FAs treatment of primary hepatocytes

**Figure 1:**
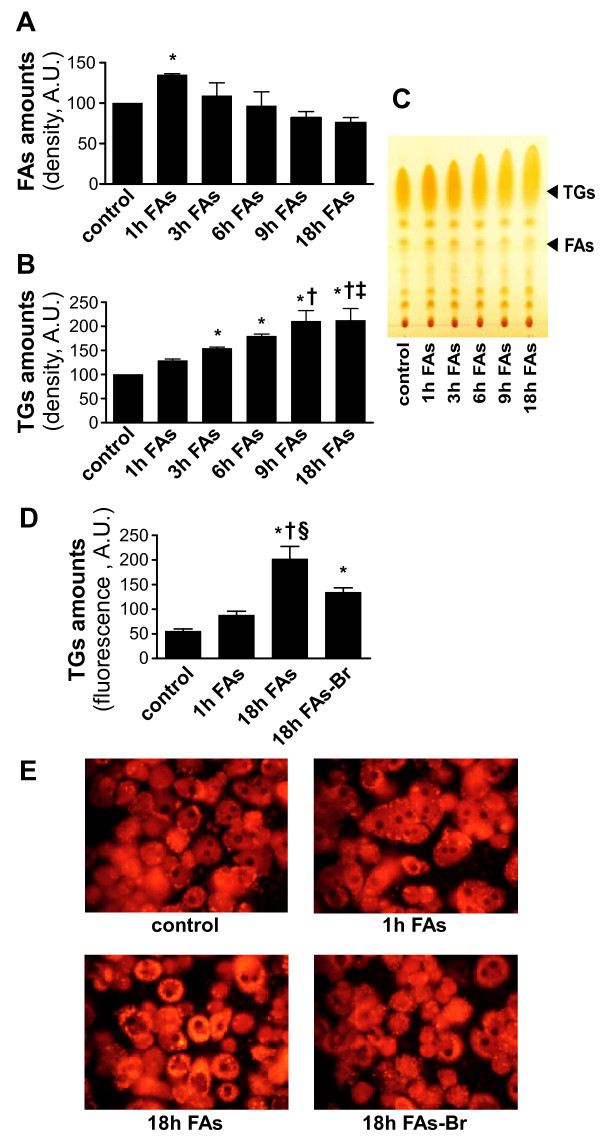
**Uptake of free-FAs and accumulation of TGs in FAs-treated cells.** Amounts of Free-FAs **(A)** and TGs **(B)** were evaluated by thin-layer chromatography in cellular extracts of primary hepatocytes cultured with FAs mixture (2 : 1 oleate:palmitate with 1% BSA) to final concentration of 1 mM for the indicated times. Quantification of lipid spots was performed by scanning densitometry. The bars represent the means ± SD of the values, relatively to the corresponding control treatment, which was normalized to 100. n = 4; Comparisons were performed using Tukey’s honestly significant differences (HSD) test. *P < .05 vs control cells cultured without FAs; †*P* < .05 vs cells cultured with FAs for 1 hour; ‡ *P* < .05 vs cells cultured with FAs for 3 hours. **(C)** A representative silica gel plate separation of cellular lipid extractions is shown, with indication of TGs and free-FAs spots position. **(D)** Primary hepatocytes were cultured and treated with FAs mixture (2 : 1 oleate:palmitate with 1% BSA) or with FAs-Br mixture (2 : 1 oleate:2-Bromopalmitate with 1% BSA) to final concentration of 1 mM FAs for the indicated times. After that, cells were stained with Nile-Red and fluorescence was measured by FACS. *P < .05 vs control cells cultured without FAs; †*P* < .05 vs cells cultured with FAs for 1 hour; § *P* < .05 vs cells cultured with FAs-Br for 18 hours. **(E)** After the indicated treatments, Nile-Red stained cultures were examined under fluorescence microscopy (magnification × 400). Yellow fluorescence indicates intracellular accumulation of TGs.

Rapid uptake of FAs by cultured primary hepatocytes is indicated by TLC separation of cellular lipid extracts. Eminent increase in cellular FAs amounts is noticed after 1 hour exposure to FAs (Figure 1A). Longer exposure to free-FAs allows their esterification into triglycerides (TGs) by the cells, as indicated by time-dependent cellular accumulation with TGs (Figure 1B and 1C). Indeed, as exposure of cells to FAs is elongated beyond 1 hour, free-FAs amounts decrease back to control levels (Figure 1A and 1C), parallel to the increase in TGs amounts.

As seen also by FACS analysis after Nile-Red staining (Figure 1D), TGs accumulation is not evident after exposure to FAs for 1 hour. Yet, as expected, massive increase in cellular TGs is noticed in cells after 18 hours exposure to free-FAs (Figure 1D). Similar results were observed when Nile-Red stained cells were examined under fluorescence microscopy (Figure 1E).

Replacement of palmitate acid with the non-metabolic FA 2-bromopalmitate (FAs-Br) markedly prevented the TGs accumulation observed after 18 hours (Figure 1D and 1E, right lower panel). 2-bromopalmitate is not esterified into neutral lipids and inhibits esterification of endogenous FAs [10] and therefore prevents TGs synthesis in the cells.

### Free-FAs inhibit gluconeogenesis induction

Based on the results presented in Figure 1, the effect of free-FAs on gluconeogenesis induction was investigated in primary hepatocytes by pre-treatment with FAs for 1 hour, prior to gluconeogenic stimuli.

**Figure 2:**
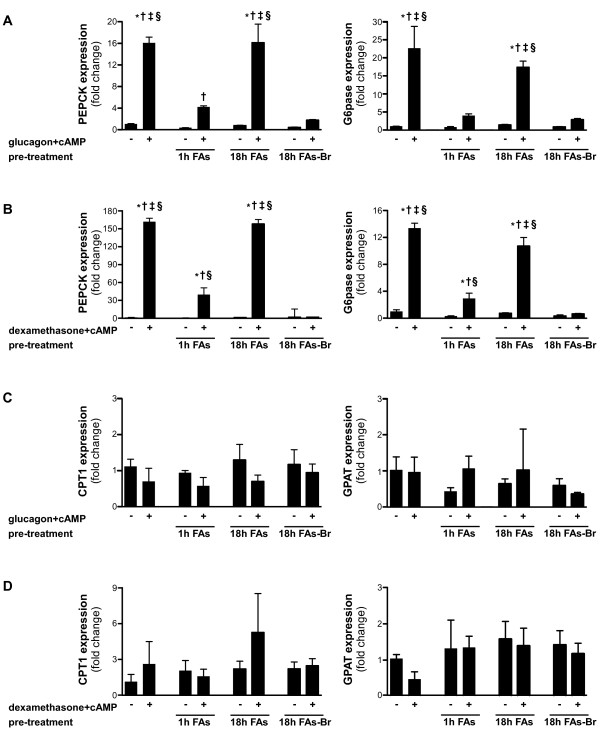
**Effect of FAs on induction of gluconeogenic genes and expression of lipid metabolism-related genes.** Primary hepatocytes were cultured and pre-treated with FAs mixture (2 : 1 oleate:palmitate with 1% BSA) or with FAs-Br mixture (2 : 1 oleate:2-Bromopalmitate with 1% BSA) to final concentration of 1 mM FAs for the indicated times. After pre-treatments, cells were treated with the gluconeogenic inducers glucagon and cAMP **(A** and **C)** or dexamethasone and cAMP **(B** and **D)** for 6 hours. mRNA expression levels of phosphoenolpyruvate carboxykinase (PEPCK), glucose-6-phosphatase (G6pase), carnitine palmitoyltransferase 1 (CPT1), and glycerol-3-phosphate acyltransferase (GPAT) were measured by quantitative real-time RT-PCR. Data are expressed as means ± SD, presented relatively to basal expression level (of cells not pre-treated with FAs nor treated with gluconeogenic inducers), which was normalized to 1. n = 3; Comparisons were performed using Tukey’s honestly significant differences (HSD) test. *P < .05 vs basal expression level; †*P* < .05 vs respective control treatment (of cells not treated with gluconeogenic inducers); ‡*P* < .05 vs cells pre-treated for 1 hour with FAs and then treated with gluconeogenic inducers; §*P* < .05 vs cells pre-treated for 18 hours with FAs-Br and then treated with gluconeogenic inducers.

Treatment of the cells with glucagon and cAMP for 6 hours induces a 16-fold increase in phosphoenolpyruvate carboxykinase (PEPCK) mRNA expression level (Figure 2A, left). PEPCK expression level is of main importance in gluconeogenesis regulation, and therefore is expected to be increased by this stimulus. As seen in Figure 2A (left), pre-treatment with FAs for 1 hour, prior to glucagon and cAMP, significantly decreases this PEPCK induction. However, 18 hours pre-treatment with FAs, which enables their esterification into TGs, has no effect on PEPCK induction by glucagon and cAMP. Accordingly, when cells are exposed to FAs-Br, which inhibits TGs synthesis and therefore allows more free-FAs, PEPCK induction is inhibited, even after 18 hours of pre-treatment.

Similar effects of free-FAs on PEPCK expression levels are seen when gluconeogenesis is induced by dexamethasone and cAMP (Figure 2B, left).

In addition, pre-treatment with FAs for 1 hour prior to glucagon and cAMP abolishes the induction of glucose-6-phosphatase (G6pase) expression in primary hepatocytes (Figure 2A, right). Like PEPCK, G6pase participates in gluconeogenic rate control in the liver. This inhibitory effect is also observed when cells are pre-treated with FAs-Br, but not with FAs, for 18 hours before gluconeogenic induction (Figure 2A, right). Likewise, when induced by dexamethasone and cAMP, G6pase expression is effected by the pre-treatments in the same manner (Figure 2B, right).

As seen in Figure 2C and D, none of the treatments altered the expression of carnitine palmitoyltransferase 1 (CPT1), the rate-limiting enzyme in long-chain FAs β-oxidation, and of glycerol-3-phosphate acyltransferase (GPAT), the rate-limiting enzyme in TGs synthesis.

**Figure 3:**
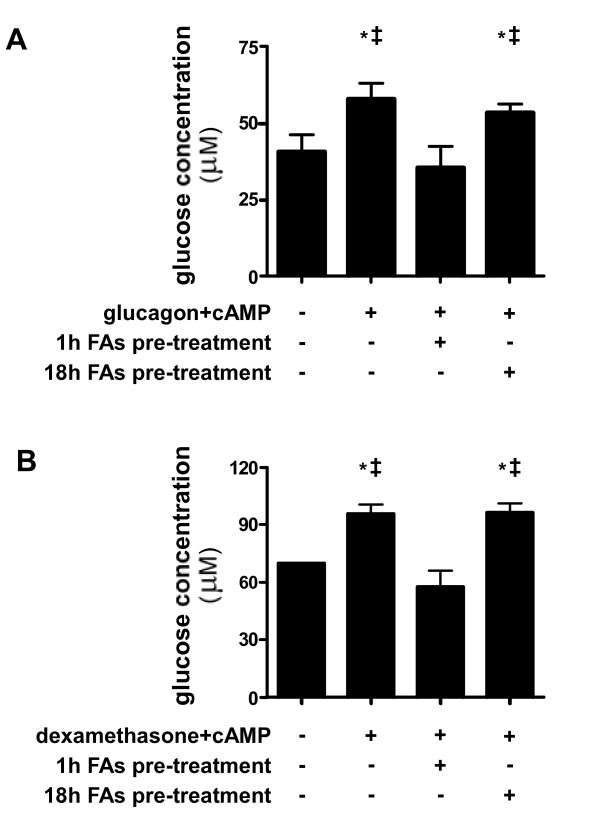
**Inhibition of induced glucose release by FAs.** Primary hepatocytes were cultured and pre-treated with FAs mixture (2 : 1 oleate:palmitate with 1% BSA) to final concentration of 1 mM FAs for the indicated times. After pre-treatments, cells were treated with the gluconeogenic inducers glucagon and cAMP **(A)** or dexamethasone and cAMP **(B)** for 6 hours. Glucose output was determined by glucose concentration measured in medium, which initially contained gluconeogenic precursors but no glucose. Glucose levels were normalized per million cells and are expressed as means ± SD. n = 3; Comparisons were performed using Tukey’s honestly significant differences (HSD) test. *P < .05 vs basal output level; ‡*P* < .05 vs cells pre-treated for 1 hour with FAs and then treated with gluconeogenic inducers.

Since both PEPCK and G6pase are rate-limiting enzymes in the gluconeogenic pathway, the inhibition of their induction represent the ability of free-FAs to inhibit induction of GNG in hepatocytes. The inhibition of GNG by free-FAs was observed as pre-treatment with free-FAs for 1 hour, but not for 18 hours, significantly inhibited glucose production by hepatocytes, when induced by glucagon and cAMP (Figure 3A) or by dexamethasone and cAMP (Figure 3B).

### The inhibitory effect of free-FAs is mediated by attenuated peroxisome-proliferator-activated receptor-γ coactivator 1α (PGC1α) response

The similar effect of FAs upon PEPCK and G6pase expression imply an upstream target for the inhibitory effect, and therefore the effect of free-FAs on PGC1α induction was examined.

**Figure 4:**
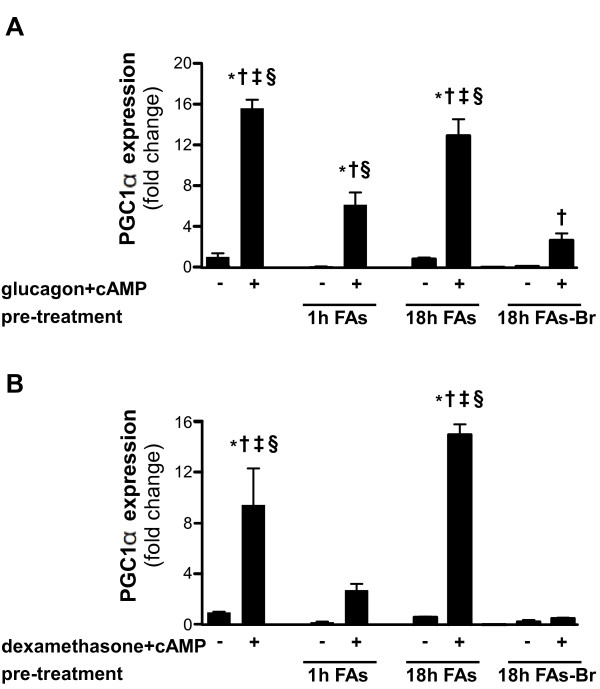
**Inhibition of PGC1α gene upregulation by FAs.** Primary hepatocytes were cultured and pre-treated with FAs mixture (2 : 1 oleate:palmitate with 1% BSA) or with FAs-Br mixture (2 : 1 oleate:2-Bromopalmitate with 1% BSA) to final concentration of 1 mM FAs for the indicated times. After pre-treatments, cells were treated with the gluconeogenic inducers glucagon and cAMP **(A)** or dexamethasone and cAMP **(B)** for 6 hours. mRNA expression levels of PGC1α were measured by quantitative real-time RT-PCR. Data are expressed as means ± SD, presented relatively to basal expression level (of cells not pre-treated with FAs nor treated with gluconeogenic inducers), which was normalized to 1. n = 3; Comparisons were performed using Tukey’s honestly significant differences (HSD) test. *P < .05 vs basal expression level; †*P* < .05 vs respective control treatment (of cells not treated with gluconeogenic inducers); ‡*P* < .05 vs cells pre-treated for 1 hour with FAs and then treated with gluconeogenic inducers; §*P* < .05 vs cells pre-treated for 18 hours with FAs-Br and then treated with gluconeogenic inducers.

As shown in Figure 4, pre-treatment with free-FAs for 1 hour results in a prominent inhibition of PGC1α expression, when induced by glucagon and cAMP (Figure 4A) or by dexamethasone and cAMP (Figure 4B). In both cases, the effect of free-FAs is diminished when pre-treatment is elongated to 18 hours, but maintained when 18 hours pre-treatment with FAs-Br is issued (Figure 4A and 4B). In fact, PGC1α expression is influenced by the pre-treatments in a similar pattern to PEPCK and G6pase, indicating that PGC1α can mediate the inhibitory effect of FAs.

### Gluconeogenic response attenuation is attributed to decreased cAMP response element-binding (CREB) phosphorylation

**Figure 5:**
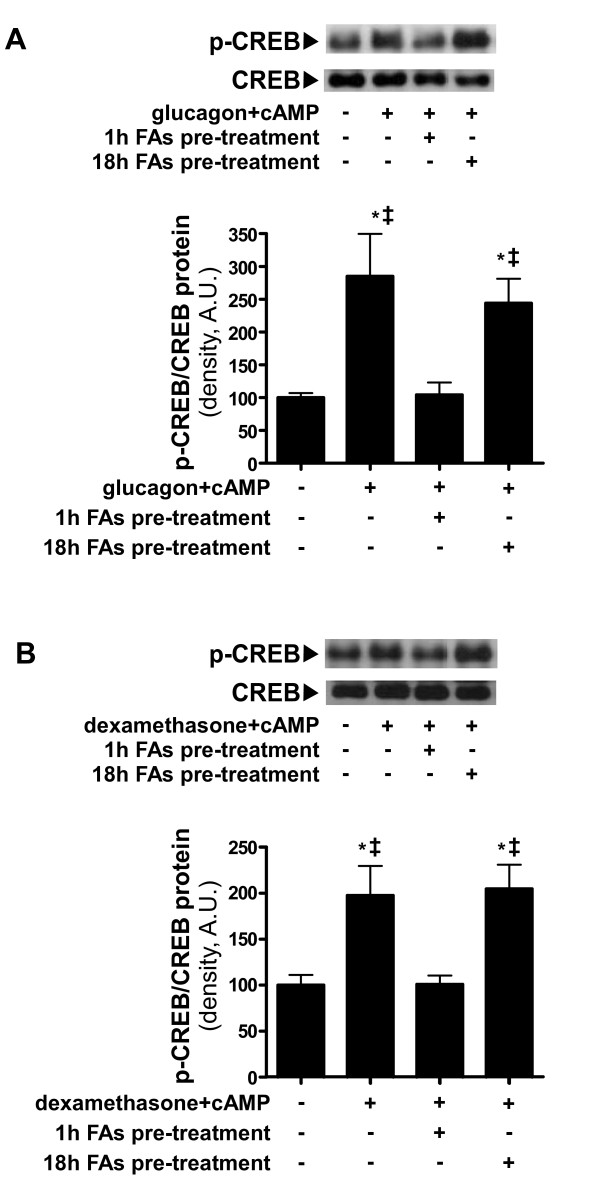
**Inhibition of CREB activation by FAs.** Primary hepatocytes were cultured and pre-treated with FAs mixture (2 : 1 oleate:palmitate with 1% BSA) to final concentration of 1 mM FAs for the indicated times. After pre-treatments, cells were treated with the gluconeogenic inducers glucagon and cAMP **(A)** or dexamethasone and cAMP **(B)** for 6 hours. CREB phosphorylation levels were measured by western-blot analysis. Quantification of bands was performed by scanning densitometry. The bars represent the means ± SD of the values, relatively to the basal expression level (of cells not pre-treated with FAs nor treated with gluconeogenic inducers) average value, which was normalized to 100. n = 3; Comparisons were performed using Tukey’s honestly significant differences (HSD) test. Representative bands are shown above bar graphs. *P < .05 vs basal expression level; ‡*P* < .05 vs cells pre-treated for 1 hour with FAs and then treated with gluconeogenic inducers.

As shown by the evaluation of p-CREB/CREB protein ratio in cellular extracts, CREB activation is induced in response to gluconeogenic stimuli of the cells with glucagon and cAMP (Figure 5A) as well as with dexamethasone and cAMP (Figure 5B). This increase in CREB phosphorylation levels is abolished by pre-treatment of the cells with FAs for 1 hour before gluconeogenic induction, but not by pre-treatment with FAs for 18 hours (Figure 5A and 5B).

Hence, inhibition of CREB activation may precede the observed effects of free-FAs on gluconeogenic response in primary hepatocytes.

## Discussion

Using an *in vitro* model of primary hepatocytes, this study provided the first evidence that free-FAs can directly attenuate the activation of gluconeogenic pathway by decreasing CREB activation. The results demonstrate that induction of glucose production and gluconeogenic enzymes is strongly inhibited by free-FAs in hepatocytes, and that the mediating link of this inhibitory effect might be inhibition of PGC1α. The inhibitory effect of free-FAs is observed when GNG is induced by different stimuli in hepatocytes, and relieved when they are esterified into TGs. Due to rapid esterification of FAs by the cells, the time window for detection of this effect is found to be short and critical. However, the duration of the effect of free-FAs is extended when their esterification is prevented by using non-metabolic FAs. Together, this implies a mechanism by which GNG is suppressed physiologically when FAs-metabolism takes over during long term fasting *in vivo*. Furthermore, exceptionally high levels of free-FAs found in the milieu of the β-oxidation deficient liver can directly attenuate GNG and therefore facilitate hypoglycemia among patients.

Although a role for FAs in modulation of GNG was previously suggested, it remained controversial whether FAs affect hepatic glucose production and how [11]. Some findings imply that free-FAs increase hepatic GNG in both healthy and diabetic individuals [12,13], as well as in the perfused liver [14,15], and in isolated hepatocytes [16,17]. Yet, the mechanism underlies this proposed stimulatory effect is far from being elucidated. Due to correlation between lipid utilization and hepatic GNG rates [16,18], the effect was mostly attributed to increased FAs oxidation. Other data demonstrate that FAs activate mitochondrial transport of pyruvate independently of their oxidation [14], thus rule out that acetyl-CoA and NADH levels are involved. Alternatively, FAs oxidation was suggested to directly stimulate gluconeogenic enzymes [19].

In contrast, an inhibitory effect of FAs on hepatic GNG has also been demonstrated both *in vitro*[20] and *in vivo*[21]. Another study reported stimulatory as well as inhibitory effects of FAs on glucose production in hepatocytes, depending upon the type of FAs and the type of gluconeogenic precursor used [22]. Therefore, some of the inconsistency could arise from the diversity of FAs, different exposure times, and variety of experimental models used in various studies. Another explanation to some of controversy might be that FAs activate GNG only in the fed state, but not under fasting [16] or when GNG is induced [23], and may even attenuate GNG during fasting [24].

Our findings, indicating an inhibitory effect of FAs on PEPCK expression in hepatocytes, are seemingly not compatible with several former data. Those include studies reporting stimulation of PEPCK expression by short-chain FAs in hepatoma cells and in primary hepatocytes, as well as by mid-, and long-chain FAs in primary hepatocytes [17,25]. Stimulatory effect was also demonstrated by oleate in FaO hepatoma cell line [26], although later experiments have failed to reproduce this result in other hepatoma cell lines or in primary hepatocytes (reported in [27]). Anyhow, performed in absence of gluconeogenic stimulus, all these previous studies reflect the effect of FAs on basal PEPCK expression rather than the effect under inductive conditions which was examined in the current study. The current data indicate that free-FAs robustly attenuate the gluconeogenic stimuli-induced PEPCK expression in hepatocytes. Since there is no known post-translational regulation of PEPCK [27], inhibition of gene induction is expected to be directly reflected in reduction of enzyme activity.

G6pase was also previously investigated by a number of studies as an optional site for FAs involvement in GNG regulation, yet much discrepancy exists between different *in vitro* and *in vivo* findings of FAs effect on G6pase expression [28]. Stimulatory effect upon G6pase expression was reported by short-chain FAs in primary hepatocytes and hepatoma cell cultures, and by mid- and long-chain FAs in isolated hepatocytes [17,29]. Another study found no effect of saturated and monounsaturated FAs, but a suppression effect of G6pase expression by polyunsaturated FAs in hepatoma cells [30]. Yet again, performed in a hormone-free environment, these studies are not representative of FAs effect when GNG is physiologically induced *in vivo*.

It should be noted that, as seen in Figure 2, free-FAs also create a small, not significant, decrease in basal PEPCK and G6pase expression (of cells not stimulated with gluconeogenic inducers). Still, the physiological importance of this observation is unclear, since basal expression levels are very low relatively to the induced expression levels. An inhibitory effect of FAs under conditions of GNG activation is in line with findings indicating suppressive effect of FAs on GNG in the fasting state *in vivo*[24].

Since FAs are metabolized by hepatocytes, their effect could be attributed to FAs *per se*, as well as to various FAs-derived metabolites, acting as a relay. Yet, the rapid effect demonstrated, obtained after 1 hour pre-treatment with FAs, does not support the idea of metabolite-mediated effect. In addition, the inhibitory effect obtained using FAs-Br, containing the nonmetabolized FA 2-bromopalmitate, is no lesser than the inhibition caused by metabolized FAs. 2-bromopalmitate is not a substrate for β-oxidation or TGs synthesis, and virtually does not incorporated into neutral lipids but rather inhibits FAs metabolism [31]. This implies that the effect is generated by FAs themselves. Nevertheless, a quick indirect effect, mediated by a FAs metabolite(s), cannot be completely excluded.

Unlike key gluconeogenic enzymes, expression levels of main lipid metabolism-related genes CPT1 and GPAT were not affected. These results are in agreement with previous studies indicating that long-chain FAs have no stimulatory effect on CPT1 gene expression in primary hepatocytes [32] and that the response of GPAT gene expression to fasting and refeeding is regulated by insulin [33]. They imply that the inhibitory effect of free-FAs upon the induction of gluconeogenic genes is direct, rather than created as a secondary result to changes induced in lipid metabolism pathways. As both PEPCK and G6pase are rate-limiting enzymes of GNG, inhibition of their induction by free-FAs resulted in elimination of the cellular gluconeogenic response, reflected by glucose release capacity of the cells (presented in Figure 3).

Additional factor that is found to exhibit the same inhibitability toward free-FAs as PEPCK and G6pase genes is PGC1α induction. PGC1α is a transcriptional coactivator, involved in expression regulation of multiple energy metabolism-related genes in the liver. When induced by gluconeogenic stimuli, PGC1α activates the entire programme of gluconeogenic genes, including PEPCK and G6pase [34], probably by interaction with HNF-4 and the glucocorticoid receptor [2]. Therefore, FAs can inhibit the induction of gluconeogenic genes by downregulating the PGC1α response.

Finally, the current findings demonstrate that the induced phosphorylation of CREB is inhibited by free-FAs. Activated by phosphorylation at Ser 133, CREB is a transcription factor which binds selectively to the consensus cAMP responsive element (CRE) sequence found in target genes promoters [35,36]. p-CREB induces PGC1 gene trough CRE element found in −130 position to transcription starting point [37]. Moreover, CRE elements are found in both PEPCK [38] and G6pase [39,40] genes and were shown to mediate the cAMP responsiveness of these promoters. Therefore, the decreased CREB activation can inhibit induction of gluconeogenic genes both directly as well as via reduced PGC-1α expression.

Several transcription-related proteins have been suggested to mediate effects of FAs on genes expression [41], while modification of transcription factors phosphorylation to alternate transactivation capacity is one proposed mechanism [42]. Interestingly, FAs were shown to stimulate basal GNG by PGC-1α and CREB in isolated hepatocytes [17], supporting the involvement of these activators in the inhibitory effect upon induced GNG. To our knowledge, the inhibition of CREB by FAs had not been reported hitherto.

## Conclusions

In summary, the presented results identify a critical role for free-FAs as inhibitors of induced GNG, which is mediated by influencing PGC1α and CREB-related signaling. By directly attenuating gluconeogenic activation in the β-oxidation deficient liver, high levels of free-FAs can promote hypoglycemia. This hypothesis suggests a novel mechanism that underlies this metabolic aberration.

## Methods

### Animals

Rats were purchased from Harlan Laboratories and were kept under standard conditions with 12 hours light/dark cycles and free access to food and water. All procedures were performed in accordance with the institutions’ guidelines of animal care.

### Primary hepatocytes isolation and culturing

Hepatocytes were isolated according to the method described [43], with minor modifications. Briefly, after anesthesia, rat livers were perfused with Hanks’ balanced salt solution (HBSS) containing 1 mM EGTA, followed by perfusion with 0.05% collagenase (cat.LS004177, Worthington, Lakeswood, NJ) in HBSS in a recirculating matter. The liver was then detached and filtered through a 70 μm nylon mesh and cells were sedimented by centrifugation. Cells were plated onto six-well plates (700,000 cells/ml) and grown in low glucose Dulbecco’s modified Eagle’s medium (DMEM) supplemented with 10% fetal calf serum, 1% L-glutamine, 100 μg/ml penicillin, and 100 μg/ml streptomycin, when kept at 37°C in humidified atmosphere (95% air and 5% CO_2_).

### Preparation of FAs

Bovine serum albumin (BSA)-complexed FAs were prepared as described [44]. FAs were dissolved in ethanol at a concentration of 100 mM. Then BSA solution (20%, w/v) was heated to 45°C, and FAs solution was gradually added to the BSA solution to achieve a complexed FAs stock solution (8 mM).

### Induction of FAs stress and GNG in primary hepatocytes

4–6 hours after plating, culture medium was replaced and cells were pre-treated with 1 mM FAs mixture of BSA-complexed palmitic acid and oleic acid (1:2) for up to 18 hours [45]. In some experiments, BSA-complexed palmitic acid in the mixture was replaced with BSA-complexed 2-bromopalmitate (FAs-Br treatment). Control cells were treated with appropriate vehicle solution (composed of BSA and ethanol).

Gluconeogenic stimuli was induced by 0.1 μM glucagon (cat.G2044, Sigma), or 1 μM dexamethasone (cat.D4902, Sigma), and 1 μM Bt2cAMP (cat.D0627, Sigma) added to the culture medium for 6 hours [34,46].

### Detection of intracellular triglycerides (TGs) accumulation

Nile-Red (cat.N3010, Sigma) stock solution (1 mg/mL) was prepared in dimethyl sulfur oxide and kept at −20°C. Cells were washed with phosphate-buffered saline (PBS), pH 7.4, and then incubated for 10 minutes in 37°C with Nile-Red solution, freshly prepared in PBS at a concentration of 1 μg/mL. Next, cells were washed again with PBS and examined by fluorescence microscopy with excitation at 540 nm and emission at 605 nm (Eclipse TS100 with Epi-fluorescence Attachment; Nikon, Tokyo, Japan) and measured by flow cytometry with excitation at 488 nm and emission at 575 nm (FACSCalibur; Becton Dickinson, CA, USA)

### Extraction of lipids from cells

Cultured cells were scraped, centrifuged, resuspended in PBS, and sampled for determination of protein concentration by Bradford method (cat.B6916, Sigma). Cells were centrifuged again, resuspended in chloroform:methanol (2:1), and shacked gently for 30 minutes. Distilled water (20%, v/v) was added and phases separation was accomplished by 15 minutes incubation at room temperature, followed by centrifugation for 15 min at 470 × g (4°C). Chloroform phase containing lipids was collected and vacuum-dried. Pellets were redissolved in chloroform for further analysis.

### Thin-layer chromatography (TLC)

Lipid extraction samples were analyzed relative to standards on a silica gel plate (Merck, Germany), as described [47]. The solvent system comprised petrol ether, diethyl ether, and acetic acid in a volumetric ratio of 80 : 19 : 1. Visualization of the compounds on the plates was performed with iodine staining. Quantitative determination of lipid spots intensity was performed by scanning densitometry.

### Total RNA isolation and quantitative real-time RT-PCR analysis

Total RNA was isolated from the cells by using Trizol according to the instructions of the manufacturer. cDNA was generated by reverse transcription of 1 μg of total RNA using High Capacity cDNA Reverse Transcription Kit (cat.4368814, Applied Biosystems, Foster City, CA). The expression level of mRNA was quantified with real-time PCR by using Sybr Green qPCR mix in a 7300 Real-Time PCR System (Applied Biosystems, Foster City, CA). The results were normalized to the Glyceraldehyde 3-phosphate dehydrogenase (GAPDH) expression (used as endogenous control) and fold-change expression was calculated by using Ct values in comparison with experimental controls that received a value of 1. Primer sequences used are reported in Table 1.

**Table 1:** Primers used for real-time PCR

**Primer name**	**Oligonucleotide sequence (5’-3’)**
PEPCK for.	ACGTGGCTGAGACAAGTGAT
PEPCK rev.	GGAAGTGATGGTGACTCCTG
G6pase for.	ATGTCTACCCGGCTTCAGTT
G6pase rev.	CACGGGCTGGTCTATCATTA
CPT1 for.	CTGCTGTATCGTCGCACATT
CPT1 rev.	GAATGGTGGATCCCAGAAGA
GPAT for.	AACGCTGAAATGGAAGGAGA
GPAT rev.	TCTGAGGCGTGCATGAATAG
PGC1α for.	GCTATGAAGCCAATGAGCAC
PGC1α rev.	TCAGACTCCCGCTTCTCATA
GAPDH for.	ATGATTCTACCCACGGCAAG
GAPDH rev.	CTGGAAGATGGTGATGGGTT

### Glucose production assay

Culture medium was replaced with 0.4 ml of glucose production medium consisting of glucose-free DMEM (pH 7.4), without phenol red, supplemented with 20 mM sodium lactate and 2 mM sodium pyruvate [34]. After 1 hour the buffer was collected and the glucose concentration was measured by enzymatic colorimetric glucose assay.

### Western blot analysis

Cells were scraped and lysed on ice in 100 μl ice-cold RIPA buffer [50 mM Tris–HCl, pH 7.4, 150 mM NaCl, 4 mM EDTA, 1% triton-X, 0.1% SDS, 1% sodium deoxycholate, 2 mM sodium orthovanadate, 5 mM NaF] with freshly added 1 mM PMSF and protease inhibitor cocktail (cat.P8340, Sigma). Lysates were transferred through 23 G needles, incubated on ice for 30 minutes, vigorously vortexed and centrifuged for 15 min at 12000 × g (4°C). Supernatants were collected and used for Western blot analysis.

Determination of total protein concentrations was carried out by the DC Protein Assay Reagents (cat.500-0116, Biorad, Hercules, CA). Protein expression was analyzed by standard Western blot techniques. In brief, protein samples were denatured in electrophoresis buffer (cat.S3401, Sigma), at 95°C for 5 minutes and equal amounts of protein were subjected to sodium dodecyl sulfate–polyacrylamide gel electrophoresis (SDS-PAGE). Proteins were transferred onto nitrocellulose membranes, and equal loading was confirmed by ponceau red staining. The membranes were blocked in Tris-buffered saline with 0.1% Tween 20 (TBS-T) containing 5% nonfat dry milk, or 5% BSA (in the case of CREB and p-CREB, accordingly) for 1 hour at room temperature. Immunodetection was done by using anti-CREB antibody (cat.9197, Cell Signaling, Danvers, MA) or anti-p-CREB antibody (cat.9198, Cell Signaling, Danvers, MA), diluted 1 : 1000 in TBS-T with 5% milk or 5% BSA, accordingly, at 4°C overnight. After a subsequent washing step, peroxidase-conjugated anti-rabbit immunoglobulin (cat.111-035-003, Jackson laboratories, West Grove, PA) was used as a secondary antibody (diluted 1 : 3500 in TBS-T with 2.5% milk at room temperature for 1 hour). Visualization of immunoreactive bands was performed by using ECL detection reagents, and the signal was detected by short exposure to x-ray film. Bands were quantified by scanning densitometry and expressed as arbitrary units. Expression levels of p-CREB in each sample were normalized to their respective CREB levels.

### Statistical analysis

The results are the means ± S.D of experiments performed in triplicate. Each experiment was repeated in cells of at least two independent cell isolation procedures. Analysis was performed by ANOVA using JMP Software (SAS Institute, Cary, NC, USA). Comparisons between groups were performed using Tukey’s honestly significant differences (HSD) test. Differences with a value of P < 0.05 were considered to be statistically significant.

## Abbreviations

FAs: Fatty acids; GNG: Gluconeogenesis; BSA: Bovine serum albumin; TLC: Thin-layer chromatography; GAPDH: Glyceraldehyde 3-phosphate dehydrogenase; TGs: Triglycerides; PEPCK: Phosphoenolpyruvate carboxykinase; G6pase: Glucose-6-phosphatase; CPT1: Carnitine palmitoyltransferase 1; GPAT: Glycerol-3-phosphate acyltransferase; PGC1α: Peroxisome-proliferator-activated receptor-γ coactivator 1α; CREB: cAMP response element-binding.

## Competing interests

The authors declare that there are no competing interests.

## Authors’ contributions

OT and ZM conceived the experimental design and performed the proofreading of manuscript. NBH performed the experiments and the statistical analysis and wrote the manuscript. SA conducted the FACS analysis. All authors discussed analyses and interpretation, read and approved the final manuscript.
